# Experience on the first national anti-TB drug resistance survey (DRS) in Timor-Leste

**DOI:** 10.1186/s41256-022-00249-z

**Published:** 2022-05-20

**Authors:** Constantino Lopes, Debashish Kundu, Ismael Da Costa Barreto, Bernardino da Cruz, S. Siva Kumar, Sureshbabu Ramalingam, Anna S. Dean, Vineet Bhatia, Prabhu Seenivasan, C. Padmapriyadarsini, Olga Tosas Auguet

**Affiliations:** 1 National TB Programme, Ministry of Health, Dili, Timor-Leste; 2 World Health Organization Country Office for Timor-Leste, Dili, Timor-Leste; 3 National Health Laboratory, Ministry of Health, Dili, Timor-Leste; 4 National HIV Programme, Ministry of Health, Dili, Timor-Leste; 5 National Institute for Research in Tuberculosis, Chennai, India; 6 National TB Reference Laboratory, Dili, Timor-Leste; 7 Global Tuberculosis Programme, World Health Organization, Geneva, Switzerland; 8 World Health Organization South East Asia Regional Office, New Delhi, India

**Keywords:** Anti-TB Drug Resistance Survey, Rifampicin resistance, Whole genome sequencing, Timor-Leste

## Abstract

**Background:**

A national drug resistance survey (DRS) was implemented for the first time in Timor-Leste (TL) in 2019. The primary objective of the survey was to assess the prevalence of drug resistance among new and previously treated pulmonary TB patients in the country.

**Methods:**

This nation-wide cross-sectional survey was conducted in 2019 targeting all new and previously treated sputum smear-positive pulmonary TB patients. Sputum samples were submitted to the National TB Reference Laboratory for confirmation of TB and to determine resistance to rifampicin by Xpert MTB/RIF. Culture was performed on solid media, and culture isolates of confirmed TB cases were shipped to the WHO Supranational TB Reference Laboratory in Chennai, India for whole genome sequencing (WGS). Survey summary statistics, data cross-tabulations and analysis of potential risk factors of rifampicin-resistant TB (RR-TB) were conducted using R statistical software (version 3.5.2).

**Results:**

A total of 953 sputum-smear positive patients were enrolled, of which 917 were confirmed as positive for TB by either Xpert MTB/RIF or culture. An electronic web-based system was used for entry and storage of the data. Rifampicin resistance was detected among 0.6% (95% CI 0.2–1.3) of new cases and 2.7% (95% CI 0.5- 8.2) of previously treated cases. WGS was conducted for validation purposes on 65 randomly selected isolates (29% of RR-TB (2/7) and 7% of RS-TB (63/910) by Xpert MTB/RIF or pDST). The original test results agreed with the WGS validation results for 62/64 isolates (97%).

**Conclusion:**

The prevalence of RR-TB in Timor-Leste is relatively low compared to the estimated proportions of RR-TB in the WHO South-East Asia Region (2.5% [95% CI 1.9–3.3] among new cases and 14% [95% CI 7.7–21] among previously treated cases). The rapid sputum collection and transportation mechanism implemented in the survey demonstrates its feasibility in low resource settings and should be replicated for routinely transporting TB specimens from microscopy labs to GeneXpert sites. Establishment of in-country capacity for rapid molecular diagnostics for both first- and second-line DST is an immediate need for achieving universal drug susceptibility testing (DST) to guide appropriate patient management.

## Introduction

Timor-Leste is a low-middle income young island nation with a population of about 1.2 million and an annual population growth rate of 1.8%. The country is at the eastern end of the Indonesian archipelago and is divided into 12 municipalities and one special economic zone (Oecusse). The country has an area of approximately 15,000 km^2^, a large part of which consists of hilly and mountainous terrain which is difficult to access. The population is thus scattered and there are large distances between people’s residences and the closest TB diagnostic centre. The country has the second highest estimated tuberculosis (TB) incidence rate and highest estimated TB mortality rate in the South-East Asia Region [1, 2].

In 2018, WHO estimated a burden of 3.1% (1.1–6.1%) rifampicin-resistant (RR) TB among new cases, and 15% (7.8–23%) among retreatment TB cases for Timor-Leste [3]. Accordingly, it was estimated that 240 cases of RR-TB (a rate of 19/100,000 population) [3] are emerging annually in the country. These pre-survey estimates were model-based, and not based on country data due to the lack of a continuous surveillance system for drug-resistant (DR) TB. The number RR-TB cases that were detected and notified between 2013 and 2018 as per the WHO Global TB Reports ranged from 2 to 12 per year. In 2019, there were 9 laboratory confirmed RR-TB cases, of which 7 RR-TB cases were diagnosed during the DRS period.

Only 52% of pulmonary TB cases were bacteriologically confirmed, and 9 TB cases were confirmed as RR-TB. Furthermore, only 11% of new and relapse cases were detected using Xpert MTB/RIF as the initial diagnostic test in 2019. The WHO End TB Strategy calls for the early diagnosis of TB and universal drug susceptibility testing (DST) using molecular WHO-recommended rapid diagnostic (mWRD), a major policy shift from smear microscopy-based TB diagnosis [4]**.**

The first national DRS in Timor-Leste was conducted by the National TB Programme (NTP) and the National TB Reference Laboratory (NTRL) in 2019, with technical assistance from the World Health Organization (WHO) and the National Institute of Research in Tuberculosis (NIRT), Chennai, India (a WHO Supranational TB Reference Laboratory, SNRL).

The primary objective of the DRS was to determine the prevalence of resistance to rifampicin among new and previously treated sputum smear-positive pulmonary TB cases in Timor-Leste, to inform the planning of the programmatic management of DR-TB (PMDT), including the roll-out of the WHO-recommended all-oral shorter regimen for RR-TB [5]**,** and guide the resource needs. The secondary objectives were to describe the sociodemographic and clinical characteristics of bacteriologically-confirmed pulmonary TB patients; investigate potential risk factors for RR-TB; establish baseline data for surveillance of DR-TB in order to allow the observation of trends over time; and strengthen the routine surveillance of anti-TB drug resistance in Timor-Leste.

## Methods

### Study design and study population

The national cross-sectional study targeted all newly registered (new and previously treated) pulmonary TB patients in the country who were sputum smear-positive by microscopy. Following a one-month pilot to test all study procedures, eligible patients were enrolled into the survey over eight months (from 21st January 2019 to 5th September 2019). Clinically diagnosed TB patients (i.e. those diagnosed without bacteriological confirmation of TB by microscopy), extra-pulmonary TB patients, sputum smear-negative cases, patients who had already received more than seven days of treatment in their current treatment course, and prisoners were excluded from enrolment.

### Sampling strategy

STROBE statement was followed to include check-list items in reporting this cross-sectional study [6]. The survey comprised 100% sampling of all Community Health Centres (CHCs) in the country offering microscopy diagnosis of TB (n = 76). The enrolment period was fixed across all CHCs to ensure that the final sample was self-weighted by the TB caseload in each facility.

The sample size calculation was based on the number of new smear positive TB cases in Timor-Leste in 2017 (n = 1511) and assumed 3.3% expected prevalence of RR-TB among new cases of bacteriologically confirmed pulmonary TB (WHO Global TB Report 2018) [7]. The recommended simple random sampling formula with a finite population correction was used [8] to calculate the sample required to estimate the proportion of RR-TB to an absolute precision of 1% and a 95% level of confidence. The sample size was increased to account for 20% potential losses of samples due to testing errors or lost/contaminated samples, to a total of 846 new bacteriologically confirmed pulmonary TB cases. A fixed enrolment period of 8 months (calculated as [846/1511] *12 and allowing for one extra month to account for any potential unforeseen circumstances) was set across all CHCs to attain the required sample. All eligible previously treated cases were enrolled opportunistically at all CHCs throughout this period.

### Patient enrollment

At CHCs, all eligible patients presenting consecutively to the facility for diagnosis of pulmonary TB, were asked to produce two sputum samples (spot and morning) for smear microscopy examination following routine practice. Patients were then enrolled following informed written consent, if at least one sputum sample was positive by smear microscopy. Following the enrolment, voluntary HIV testing was conducted as per national guidelines (provider-initiated testing and counselling). Clinical, demographic and previous anti-TB treatment history data were then collected from each patient through a questionnaire (DRS Clinical Information Form [CIF]), which was completed by the TB focal person at the CHC.

### Sample collection and laboratory procedures

The two sputum samples collected from the eligible patients at CHCs were transported to NTRL, Dili, maintaining a cold chain and within 3–5 days. At NTRL, both samples again underwent sputum smear microscopy and inoculation on solid (Lowenstein Jensen) media. Morning samples were also tested by Xpert MTB/RIF according to manufacturer’s instructions. Colonies were scraped from both solid culture slants and diluted in Phosphate Buffer Solution (PBS). One PBS aliquot was kept at NTRL for back-up storage (−20 °C). A second PBS aliquot was shipped to Chennai supranational reference laboratory (SNRL) maintaining a cold chain, following the IATA regulations for biological substances, Category A infectious material (UN3373 [9]). For the purpose of validating Xpert MTB/RIF resistance test results, whole genome sequencing (WGS) was conducted at Chennai SNRL for approximately 5–10% of TB isolates with rifampicin resistance results by Xpert MTB/RIF. For isolates that were “MTB not detected” or “MTB detected with indeterminate rifampicin resistance result” by Xpert MTB/RIF, resistance to rifampicin was evaluated by phenotypic DST (pDST) in liquid culture (BACTEC MGIT), if the culture isolate was confirmed as *Mycobacterium tuberculosis* at the SNRL.

WGS was conducted using Illumina HiSeq X Ten system (Illumina, San Diego, CA, USA), following extraction of genomic DNA from the clinical isolates using the CTAB (cetyl trimethylammonium bromide) method. DNA was purified using the Genomic DNA Clean and Concentrator kit and assessed for quality and quantity using Nano Drop and Qubit dsDNA Assay kits (Thermo Fisher Scientific, Waltham, MA, USA). Libraries were prepared using NEBNext Ultra DNA Library preparation kit (Illumina, San Diego, CA, USA). For the subset of isolates tested by WGS, mutations conferring resistance to isoniazid, ethambutol, streptomycin, pyrazinamide and fluoroquinolones were also examined.

### Data management

An electronic web-based DRS database (https://tb.ms.gov.tl/tb1) [10] was designed prior to the start of the survey for the purposes of data entry and storage of the data from the DRS. The DRS database was designed to meet the requirements of confidentiality principles and was password protected and backed-up daily to a secure server. The database contained all the data fields from patient questionnaires as well as all NTRL and SNRL laboratory test results. A separate section of the database was designed for the electronic tracking of samples, shipments, and reports of laboratory results to CHCs.

### Data analysis

Survey summary statistics, data cross-tabulations and analysis of potential risk factors of RR-TB were conducted using R statistical software (version 3.5.2) [11]. For each anti-TB drug, the prevalence of resistance along with the corresponding 95% confidence intervals (95% CI), was estimated by fitting a logistic regression and then taking the inverse logit of the model coefficient and confidence interval. Resistance to rifampicin was determined by considering both Xpert MTB/RIF and WGS test results, with the latter providing a conclusive result in case of discrepancies. Associations between RR-TB and clinical and demographic traits were explored in univariate logistic regression analyses adjusted by previous anti-TB treatment history. Measures of association for potential predictors were summarised by odds ratios with the corresponding 95% confidence intervals.

## Results

### Clinical and demographic characteristics of the TB population

A total of 953 eligible patients were enrolled from 69 CHCs across 12 municipalities and one special economic zone over the study period. Seven CHCs did not report any TB patients. The median time taken from sample collection to receipt of the samples at NRTL was 2 days (interquartile range of 1–3 days). Approximately 19.3% (184/953) of the samples took more than 4 days to arrive to NTRL.

**Fig. 1:**
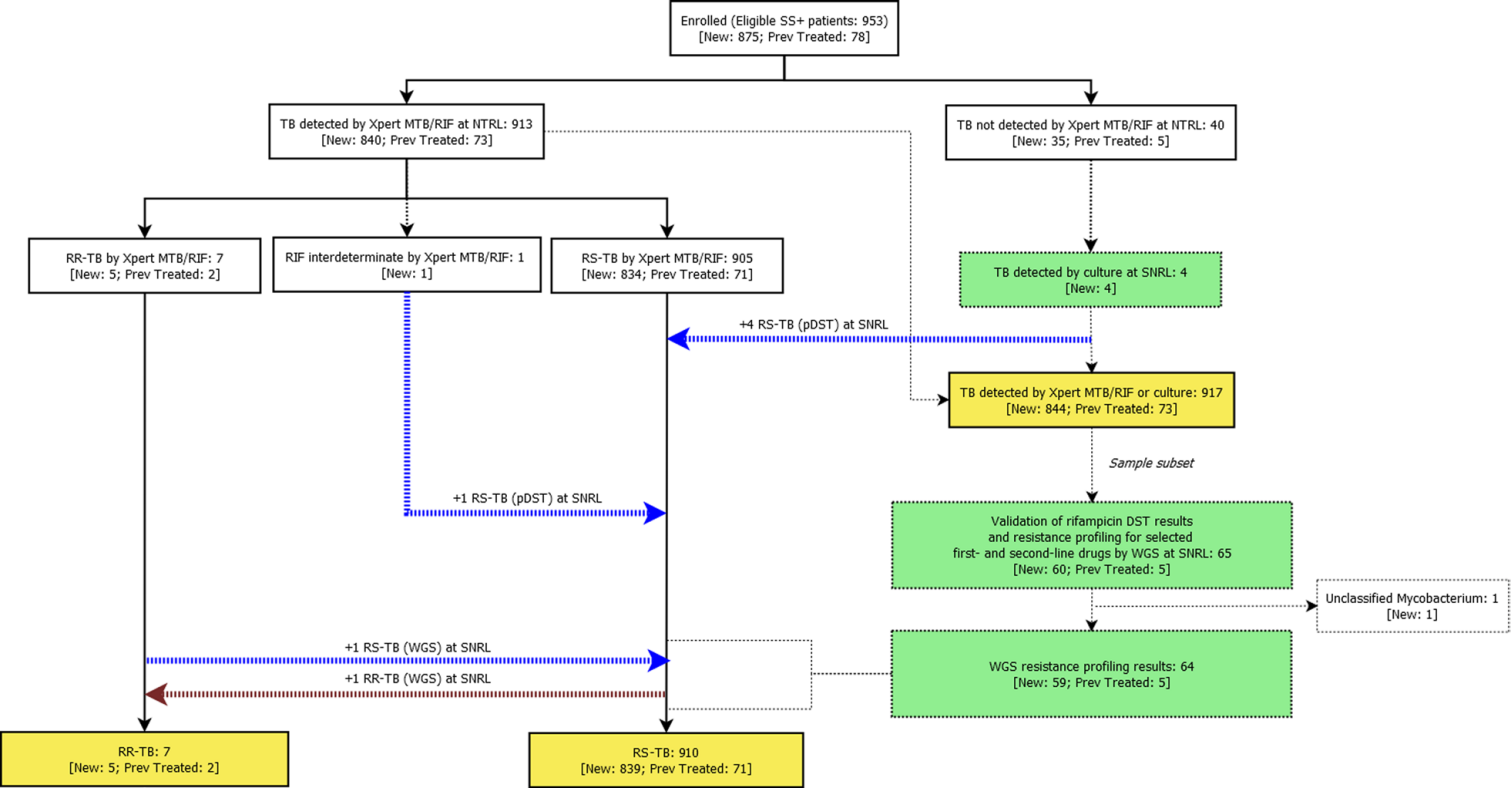
Timor-Leste’s anti-TB drug resistance survey sample processing workflow. SS +  = sputum smear-positive (on microscopy); Prev Treated = previously treated; RIF = rifampicin; RR-TB = rifampicin-resistant TB (including MDR-TB); RS-TB = rifampicin-susceptible TB; pDST = phenotypic DST; WGS = whole genome sequencing; NTRL = National TB Reference Laboratory; SNRL = Supranational Laboratory

**Fig. 2:**
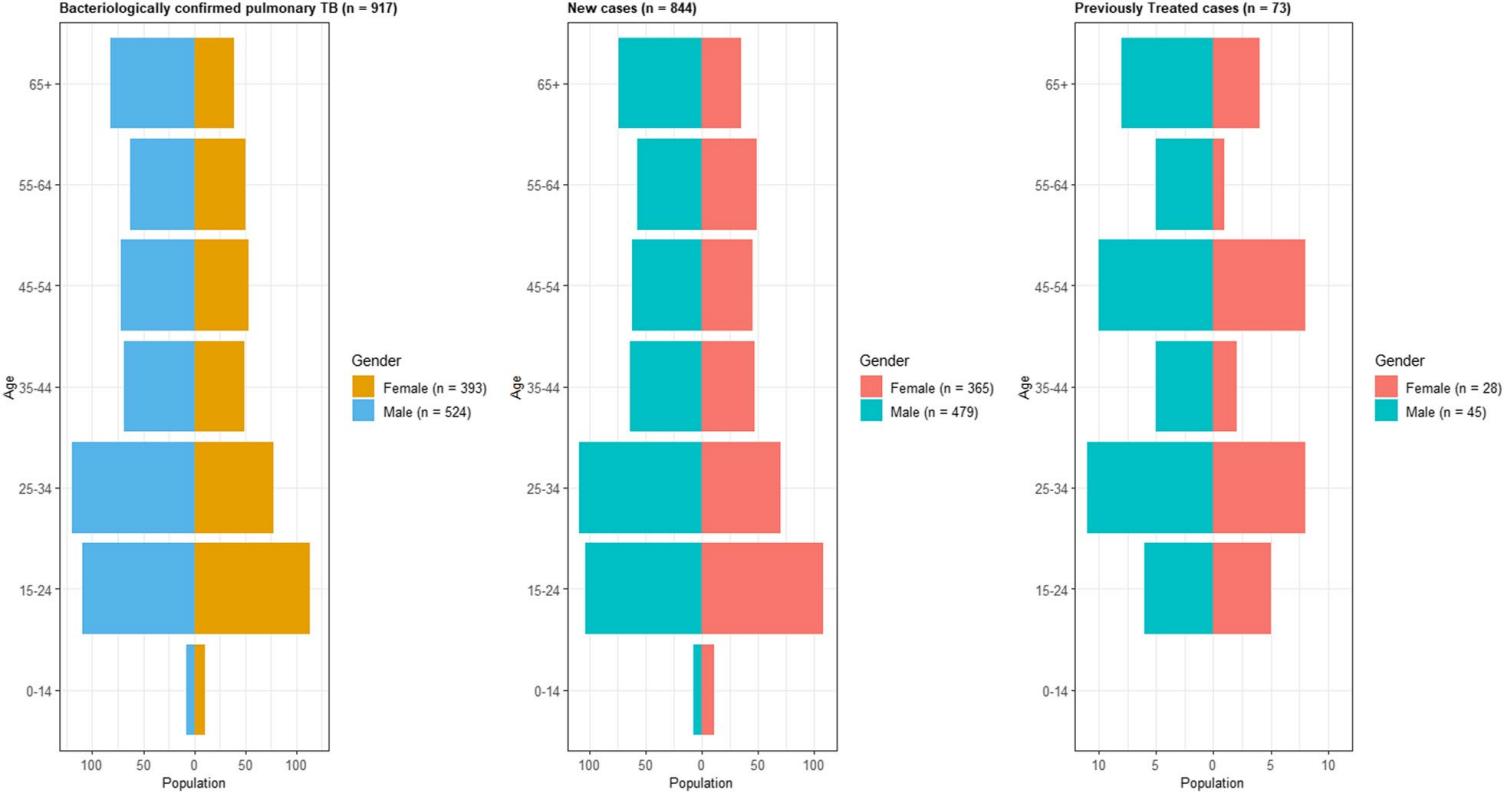
Age/sex population structure of bacteriologically confirmed pulmonary TB patients

Out of 953 enrolled patients, 917 were confirmed as pulmonary TB cases by either Xpert MTB/RIF and/or culture (Fig. 1). More than half of bacteriologically confirmed TB patients were male (57.1%), and 8.0% had been previously treated for TB (Table 1). Only 2.1% of TB cases were children aged < 15 years. More TB cases were identified from individuals aged 15–34 years (45.9%). A total of 12 (1.3%) patients were co-infected with HIV. Most cases were detected from the capital of the country, Dili, and the Ermera municipality, which together comprised 59.4% of the cases (Table 1). The age and sex population structure for bacteriologically confirmed pulmonary TB patients enrolled in the study (n = 917) is shown in Fig. 2.

**Table 1:** Demographic and clinical characteristics of bacteriologically confirmed TB patients enrolled in the survey

Patient characteristics	New N = 844		Previously treated N = 73		Combined N = 917	
	Frequency (n)	%	Number	%	Number	%
**Gender**						
Male	479	56.8	45	61.6	524	57.1
Female	365	43.2	28	38.4	393	42.9
**Age**						
0–14	19	2.2	0	0.0	19	2.1
15–24	212	25.1	11	15.1	223	24.3
25–34	179	21.2	19	26.0	198	21.6
35–44	111	13.2	7	9.6	118	12.9
45–54	107	12.7	18	24.7	125	13.6
55–64	107	12.7	6	8.2	113	12.3
65+	109	12.9	12	16.4	121	13.2
**HIV status**^**a**^						
Negative	863	98.7	77	98.6	904	98.7
Positive	11	1.3	1	1.4	12	1.3
**Municipality**						
Aileu	23	2.7	2	2.7	25	2.7
Ainaro	14	1.7	0	0.0	14	1.5
Baucau	43	5.1	4	5.5	47	5.1
Bobonaro	68	8.1	6	8.2	74	8.1
Covalima	37	4.4	4	5.5	41	4.5
Dili	297	35.2	28	38.4	325	35.4
Ermera	211	25.0	9	12.3	220	24.0
Lauten	26	3.1	5	6.8	31	3.4
Liquica	31	3.7	5	6.8	36	3.9
Manatuto	24	2.8	3	4.1	27	2.9
Manufahi	17	2.0	4	5.5	21	2.3
Oecusse	33	3.9	2	2.7	35	3.8
Viqueque	20	2.4	1	1.4	21	2.3

^a^One new TB patient with missing HIV status

### Sample processing workflow at NTRL and SNRL

Out of 953 patients enrolled into the study based on smear microscopy findings at CHCs, 913 were confirmed as pulmonary TB patients by Xpert MTB/RIF and a further four were confirmed by culture (n = 917). See Fig. 1. Out of those confirmed as pulmonary TB by Xpert MTB/RIF, seven were RR-TB and 905 were RS-TB. Rifampicin resistance was indeterminate by Xpert MTB/RIF for one case. Phenotypic DST for this indeterminate specimen, plus four isolates that were confirmed as MTB by culture, showed all five isolates were RS-TB, totaling 7 RR-TB isolates plus 910 RS-TB isolates by Xpert MTB/RIF and/or culture.

WGS was conducted for validation purposes on 65 randomly selected isolates (29% of RR-TB (2/7) and 7% of RS-TB (63/910) by Xpert MTB/RIF or pDST). Of these, one isolate was excluded due to inconclusive speciation results, resulting in non-interpretable resistance profiling (Fig. 1). The original test results agreed with the WGS validation results for 62/64 isolates (97%). WGS re-classified one RR-TB by Xpert MTB/RIF as susceptible to rifampicin, and one RS-TB by Xpert MTB/RIF as resistant to rifampicin. For the case of the isolate that was confirmed as resistant by WGS, a rare mutation associated with rifampicin resistance (Ser441Gln) was identified in the rifampicin resistance determining region (RRDR) region. This was an unusual double mutation -where two consecutive nucleotides are replaced by two different nucleotides on the genome- leading to *rpoB* 441st codon TCG being replaced by CAG (HGVS nomenclature on rpoB gene sequence: c.1321_1322delinsCA). No resistance mutations were identified in the case of the isolate that was confirmed as susceptible by WGS.

### Resistance to anti-TB drugs

The prevalence of rifampicin resistance among new cases was 0.6% (95% CI 0.2–1.3) and that among previously treated TB cases was 2.7% (95% CI 0.5–8.2). The prevalence of rifampicin resistance among these groups combined was 0.8% (95% CI 0.3–1.5). See Table 2. Of the two RR-TB with WGS test results, one also had resistance mutations for isoniazid, ethambutol and streptomycin, and a second one showed no resistance mutations to evaluated drugs (i.e. isoniazid, ethambutol, streptomycin, pyrazinamide and fluoroquinolones). Among the 64 isolates with WGS test results, no mutations conferring resistance to pyrazinamide or fluoroquinolones were identified; mutations conferring resistance to isoniazid, ethambutol and streptomycin were identified from seven, two and one isolates respectively. Due to the low testing coverage of *M. tuberculosis* by WGS (7% [64/917]), resistance estimates and corresponding 95% confidence intervals for drugs other than rifampicin were not calculated in this study. No factors were associated with RR-TB in univariate logistic regression analyses (Table 3). Multiple regression analysis was not conducted due to the small number of RR-TB cases (n = 7) identified.

**Table 2:** Prevalence of rifampicin resistance among TB patients in the survey, overall and by previous anti-TB treatment history

	Total TB cases	RR-TB	% (95% CI)
New patients	844	5	0.6 (0.2–1.3)
Previously treated patients	73	2	2.7 (0.5–8.2)
All patients	917	7	0.8 (0.3–1.5)

*RR-TB* rifampicin resistant tuberculosis, *95% CI* 95% confidence intervals

**Table 3:** Risk Factors for development of rifampicin-resistant TB

Variable	Level	Total TB caes	RR-TB cases	Odds raion (95% CI)	*p*-value
Treatment history	New	844	5		0.11
	Previously treated	73	2	4.7 (0.7–22.4)	
Gender	Male	524	4		0.96
	Female	393	3	1.0 (0.2–4.8)	
HIV status^a^	Negative	904	7		NA
	Positive	12	0	NA	
Age	15–24	223	1		0.99
	0–14	19	0	NA	
	25–34	198	2	4.5 (0.2–43.4)	
	35–44	118	1	1.8 (0.1–47.1)	
	45–54	125	1	1.4 (0.1–36.3)	
	55–64	113	1	2.0 (0.1–50.1)	
	65 +	121	1	1.6 (0.1–41.4)	
Municipality	Other municipalities	592	2		0.06
	Dili capital	325	5	4.5 (1.0–31.7)	

Univariate logistic regression analyses of key demographic and clinical variables as potential predictors of rifampicin resistance. Analyses were adjusted by treatment history. Variable levels with zero cases were excluded from the analyses; consequently, no analysis was conducted for HIV, but numbers of rifampicin resistant cases disaggregated by HIV status are included in the table for reference

^a^HIV status was missing for one patient

## Discussion

This is the first national survey of drug resistance among people with TB in Timor-Leste, providing the first reliable estimate of the burden of drug-resistant TB in the country. The survey highlighted challenges in conducting nationwide studies in resource limited settings [12]**.** The survey was not only an opportunity to collect reliable data, but also to strengthen the health system overall. The on-site training of primary health care doctors, municipality coordinators and five referral hospitals for timely and correct diagnosis, and provision of treatment and care not only resulted in smooth survey implementation but also developed capacity of health centres and diagnostic laboratory networks.

Most TB cases identified were male and under 55 years of age. Notably, children < 15 years old account for 11% of TB globally, but only represented 2% of cases in Timor-Leste during this 8-month period. The WHO Global TB Report 2020 [13] estimated that 8% of notified TB cases were children in Timor-Leste, including both clinically diagnosed and bacteriologically confirmed pulmonary and extra-pulmonary TB. This difference may be partially explained by the DRS enrolling only bacteriologically confirmed pulmonary TB cases. Obtaining bacteriological confirmation of TB in children remains a challenge.

The overall prevalence of rifampicin resistance was 0.8% (95% CI 0.3–1.5%) among all TB patients, with 0.6% (95% CI 0.2–1.3%) among new TB patients and 2.7% (95% CI 0.5–8.2%) among previously treated TB patients. This is notably lower than previous modelled estimates prior to the survey. These figures are also lower than in neighbouring Indonesia, at 2.4% (95% CI 1.8–3.3%) among new cases and 13% (95% CI 9–18%) among previously treated cases, as well as the average for the WHO Southeast Asia Region, at 2.5% (95% CI 1.9–3.3%) among new cases and 14% (95% CI 7.7–21%) among previously treated cases [13]**.** Surveys in other small countries like Lao PDR [14] and Eritrea [15] were similarly low**.** One possible explanation for these lower level of rifampicin resistance in Timor-Leste may relate to the fact that rifampicin was only introduced into the four-month continuation phase of the first-line treatment regimen from the second quarter of 2015 (in line with the WHO-recommended regimen of 2HRZE + 4RH), which was much later than in neighbouring countries. The lower level of rifampicin resistance found in this study in Timor-Leste might also be attributed to a good treatment success rate among TB patients who were enrolled on treatment (88% in new and relapse TB cases in 2018). A low prevalence of drug resistance among TB cases in the previous survey in Philippines also reflected on to the high treatment success rates and the availability of second-line therapy for patients with DR-TB [16]. However, treatment coverage is low (63%) compared to the estimated number of incident TB cases in Timor-Leste, and this also may have led to missing of TB cases with DR-TB strains. Transmission rates of TB remain high, with the second highest incidence rate (498 cases per 100,000 population) in the WHO South-East Asia Region and among the top ten in the world.

This study showed good agreement between Xpert MTB/RIF and WGS results (97%), showing that Xpert MTB/RIF performs reliably in this setting. However, WGS also identified an unusual double mutation in the RRDR region of the genome which was missed by Xpert MTB/RIF. The occurrence of mutations missed by routine diagnostic tools should be regularly monitored in the region through collaborations with supranational referral laboratories, SNRLs, for quality assurance purposes. One RR-TB case by Xpert MTB/RIF, later found to be susceptible to rifampicin by WGS, may be explained by the analysis of an heteroresistant sample by Xpert MTB/RIF and/or the preferential selection of a susceptible strain upon culturing a sputum sample harbouring a mixed infection, prior to WGS. We also found that of the 40 samples that tested negative to *M. tuberculosis* by Xpert MTB/RIF at NTRL, four were positive by culture. It is possible that the bacterial load may have been too low for detection by Xpert MTB/RIF. Similar findings have been reported in other studies [17].

Currently, eleven GeneXpert machines are functional in Timor-Leste. This is inclusive of two 16 modular GeneXpert machines, received for the COVID-19 response, which can be used by the NTP for TB diagnosis. Linkages with the GeneXpert machines will also be prioritised for intensified case finding at all facilities with a high outpatient caseload [18]. During the survey, sputum samples were transported from all the municipalities in the country to the GeneXpert sites and NTRL with minimal delay (median of 2 days) through human carriers who were adequately incentivized for this work. Delay was observed in sample shipment from NTRL, Dili, to the SNRL, Chennai, and the availability of the courier agency to ship biohazard samples, which led to decreased viability and contamination of transported samples.

The WHO End TB Strategy calls for the early diagnosis of TB and universal DST, highlighting the critical role of laboratories for rapidly and accurately detecting TB and drug resistance. Overall, the proportion of all notified TB cases routinely tested by Xpert MTB/RIF in Timor-Leste is only around 11% despite having sufficient Xpert MTB/RIF capacity in the country. To address this gap, the NTP intends to move towards universal DST by Xpert MTB/RIF as quickly as possible, as per the revised national TB guidelines (Fifth Edition, 2020) [18] to first include testing of all bacteriologically confirmed TB cases and ultimately all presumptive TB cases, translating policy into practice. Building on lessons learned during the survey, an efficient specimen referral system is being planned with the support of partners. This will help to address barriers to accessing TB diagnosis and care from remote, hilly terrains in this island nation.

Although this study did not formally evaluate resistance to drugs other than rifampicin due to the limited number of sequenced isolates, mutations conferring resistance to isoniazid, ethambutol and streptomycin were identified among the 64 randomly selected isolates on which WGS was performed, with 7 isolates displaying genotypic resistance to isoniazid. No resistance-conferring mutations for pyrazinamide or fluoroquinolones were observed among sequenced samples, which may bode well for the management of DR-TB in the country. WHO recommends the use of Line Probe Assay (LPA) as one of the rapid diagnostic test for the detection of rifampicin, isoniazid, fluoroquinolone and pyrazinamide resistance [19]. A Regional Green Light Committee (r-GLC) mission in 2018 in Timor-Leste recommended investment by the country in LPA [20], to inform appropriate patient management in a timely way. The TB Joint Monitoring Mission (JMM) conducted in Timor-Leste in 2019 also recommended that the NTP consider introducing LPA capacity into the NTRL. By mid-2021, it is anticipated that all bacteriologically confirmed TB patients will be offered DST for rifampicin and isoniazid, as well as fluoroquinolones among cases of RR-TB, thus facilitating transition to the WHO-recommended all-oral shorter regimen.

The survey had several limitations. For logistic reasons, it was limited to only sputum smear positive patients. However, there is no evidence that the prevalence of drug resistance differs according to smear results. While rifampicin testing results by Xpert MTB/RIF were available for most participants, full drug resistance profiles were only available for a subset, thus preventing an estimation of the prevalence of resistance to other drugs. The numbers of RR-TB cases identified (7 cases only), were too small to conclusively interpret risk factors.

## Conclusions

The survey highlighted the opportunity and potential for strengthening sputum specimen transportation, establishing electronic recording and reporting, achieving NTRL accreditation, and ultimately implementing universal DST in Timor-Leste for both first- and second-line drugs. The relatively low prevalence of RR-TB in Timor-Leste is an encouraging finding, but gaps remain in obtaining bacteriological confirmation of TB and routine rifampicin testing among bacteriologically confirmed cases. This study showed that the highest burden of TB was in economically productive age groups and predominantly in males, as in other countries. Thus, the survey highlights that addressing TB, including its drug resistant forms, merits enhanced and sustained investment.

## Data Availability

Authors confirms availability of data and relevant materials.
